# Assessment of the Analytical Sensitivity of 10 Lateral Flow Devices against the SARS-CoV-2 Omicron Variant

**DOI:** 10.1128/jcm.02479-21

**Published:** 2022-02-16

**Authors:** Joshua Deerain, Julian Druce, Thomas Tran, Mitchell Batty, Yano Yoga, Michael Fennell, Dominic E. Dwyer, Jen Kok, Deborah A. Williamson

**Affiliations:** a Victorian Infectious Diseases Reference Laboratory, Royal Melbourne Hospital at The Peter Doherty Institute for Infection and Immunity, Victoria, Australia; b Centre for Infectious Diseases and Microbiology Laboratory Services, NSW Health Pathology, Institute of Clinical Pathology and Medical Research, Westmead, NSW, Australia; c Department of Infectious Diseases, The University of Melbourne at the Peter Doherty Institute for Infection and Immunity, Victoria, Australia; Boston Children's Hospital

**Keywords:** COVID-19, antigen tests, diagnostics, infectious disease, Omicron

## LETTER

Timely and accurate diagnostic testing is a critical component of the public health response to coronavirus (CoV) disease 2019 (COVID-19). Antigen (Ag) tests are used widely in many countries to provide rapid, economical, and accessible point-of-care testing (1). The vast majority of antigen tests detect nucleocapsid (N) protein, a structural protein that displays less variation than the spike (S) protein across different severe acute respiratory syndrome (SARS)-CoV-2 lineages. Although antigen tests are less sensitive than reverse transcription-PCR (RT-PCR) tests, their ability to quickly detect individuals with high viral loads provides clinical and public health utility in many countries, including Australia, where antigen tests have recently been approved for self-testing (2). As new variants arise, including the recent SARS-CoV-2 Omicron variant, it is essential to rapidly assess the performance of diagnostic assays. Here, in order to assess and compare the abilities of antigen tests to detect the Delta and Omicron variants, we performed a rapid assessment of 10 commercially available antigen tests.

To evaluate analytical sensitivity, we used representative Delta and Omicron isolates cultured from clinical samples (see the supplemental material). Isolates were grown in Calu-3 cells, as described in the supplemental material. For each variant, we constructed a dilution series of quantified virus, ranging from ∼10^9^ to 10^5^ copies/mL, corresponding to cycle threshold (Ct) values of ∼18 to ∼29 from an in-house RT-PCR for the N gene (3). Testing was performed according to the manufacturers’ provided instructions for use (IFU). Where no specific protocol was provided, a 1:1 dilution of sample to extraction buffer was prepared and added to the test kit. Interpretations of antigen test results were performed as per the manufacturers’ instructions, and results of all tests were recorded independently by two scientists. All antigen testing was conducted in quadruplicate and under biosafety level 3 conditions using live virus.

**FIG 1:**
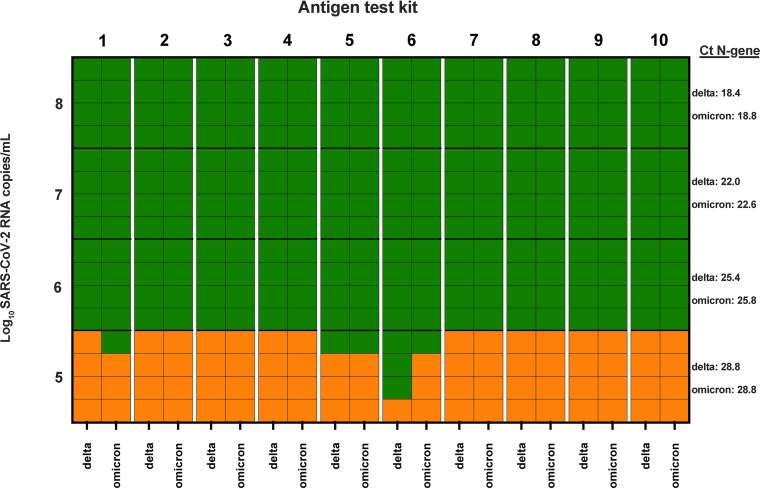
Analytical sensitivities of lateral flow devices against SARS-CoV-2 Delta and Omicron variants. Ten lateral flow devices were tested against 10-fold dilutions (1:100 to 1:100,000) of SARS-CoV-2 Delta and Omicron variants in quadruplicate. Green boxes indicate where SARS-CoV-2 antigen was detected for a single replicate, and orange boxes indicate where a negative result was observed. Mean Ct values from three replicates for each dilution were calculated using in-house RT-PCR for the N gene. The registered test names and manufacturers of the lateral flow devices included were as follows: (1) PanBio COVID-19 Ag rapid test device (nasal), Abbott Rapid Diagnostics Jena GmbH (Germany); (2) NowCheck COVID-19 antigen test, BioNote Inc. (Republic of Korea); (3) Roche SARS-CoV-2 rapid antigen test, SD Biosensor Inc. (Republic of Korea); (4) Standard Q COVID-19 Ag test, SD Biosensor Inc. (Republic of Korea); (5) SureScreen Diagnostics COVID-19 antigen rapid test cassette, BTNX Inc. (Canada); (6) VivaDiag SARS-CoV-2 Ag rapid test, VivaChek Biotech (Hangzhou) Co. Ltd. (China); (7) Wantai SARS-CoV-2 Ag rapid test (colloidal gold), Beijing Wantai Biological Pharmacy Enterprise Co. Ltd. (China); (8) Testsea SARS-CoV-2 antigen test kit, Hangzhou Testsea Biotechnology Co. Ltd. (China); (9) InnoScreen COVID-19 antigen rapid test device, Innovation Scientific Pty. Ltd. (Australia); and (10) LYHER novel coronavirus (COVID-19) antigen test kit (colloidal gold), Hangzhou Laihe Biotech Co. Ltd. (China).

Overall, the analytical sensitivities of the 10 antigen kits were similar for both the Delta and Omicron variants (Fig. 1). All 10 kits were able to detect Delta at 6.50 log_10_ copies/mL (Ct, 25.4) and Omicron at 6.39 log_10_ copies/mL (Ct, 25.8), consistent with the results of previous work on the analytical sensitivities of antigen kits (4). None of the 10 kits consistently detected either Delta or Omicron at the lowest dilutions (5.23 log_10_ copies/mL, with a Ct of 28.8 [Delta]; 5.33 log_10_ copies/mL, with a Ct of 28.8 [Omicron]).

Our results provide valuable rapid *in vitro* data on the abilities of antigen tests to detect the Omicron variant and are consistent with other work demonstrating the effectiveness of antigen tests for SARS-CoV-2 variants (5). Given the large investments made in antigen testing in many countries and the increasing reliance on these tests to inform clinical and public health action, ongoing postmarket validation, including assessment of clinical sensitivity against new variants, is essential to ensure optimal test selection and deployment.
