# Supplementary material for: Assessment of the Analytical Sensitivity of 10 Lateral Flow Devices against the SARS-CoV-2 Omicron Variant
Source: J Clin Microbiol. 2022 Feb 16;60(2):e02479-21. doi: 10.1128/jcm.02479-21 (PMC8849215; doi:10.1128/jcm.02479-21)
Supplement: Supplemental file 1 — Table S1. Download jcm.02479-21-s0001.pdf, PDF file, 0.1 MB [file jcm.02479-21-s0001.pdf]

SUPPLEMENTARY APPENDIX

Assessment of the analytical sensitivity of ten lateral flow devices against the SARS-CoV-2 omicron variant

SARS-CoV-2 cell culture

SARS-CoV-2 Delta (VIC/18440/2021) was originally isolated from residual viral transport medium from a reverse transcriptase PCR (RT-PCR)-positive swab collected in April 2021 in the Victorian infectious Diseases Reference Laboratory (GISAID Accession ID: EPI_ISL_1913206). SARS-CoV-2 Omicron (80-21-331-3676) was kindly provided by the NSW Health Pathology-Institute of Clinical Pathology and Medical Research (ICPMR), Westmead, NSW, Australia. Full genome sequencing and bioinformatic analysis an determined the lineage as B.1.1.529 (Omicron) (GISAID Accession ID: EPI_ISL_6814922). To prepare the stock used in this study, 100µL of virus isolate was added to 2 x T25 flasks of Calu-3 cells grown to 80% confluency and allowed to absorb for 30mins at 37°C. 10mL of Calu-3 infection media (DMEM/F-12, supplemented with GlutaMAX *Gibco*, 2% heat inactivated FBS) was added to each flask and cells incubated at 37°C. When CPE was observed (approximately 48-72 hours post infection) supernatant from each flask was harvested, combined and cell debris removed by centrifugation. Aliquots of virus were stored at -80°C. Quantification of the dilution series was performed by real-time RT-PCR and droplet digital PCR (ddPCR), using previously described methods (1).

Supplementary Table 1. Antigen tests evaluated in this study, with assay-specific sample and diluent volumes.

Test #	Test Name	Manufacturer	Time (mins)	Buffer volume (µL)		
				Extraction	Sample	Assay input
1	Panbio™ COVID-19 Ag Rapid Test Device (Nasal)	Abbott Rapid Diagnostics Jena GmbH (Germany)	15	150	150	130
2	NowCheck COVID-19 Antigen Test	BioNote Inc (Korea - Republic of)	15-30	300	300	50
3	SARS-CoV-2 Rapid Antigen Test	SD Biosensor Inc (Korea - Republic of)	15-30	300	350	50
4	STANDARD™ Q COVID-19 Ag Test	SD Biosensor Inc (Korea - Republic of)	15-20	300	300	50
5	Surescreen Diagnostics COVID-19 Antigen Rapid Test Cassette	BTNX Inc (Canada)	15	300	300	80
6	VivaDiag™ SARS-CoV-2 Ag Rapid Test	VivaChek Biotech (Hangzhou) Co Ltd (China)	15-20	300	300	60
7	Wantai SARS-CoV-2 Ag Rapid Test (Colloidal Gold)	Beijing Wantai Biologicalpharmacy Enterprise Co Ltd (China)	20-30	60	300	100
8	Testsea SARS-CoV-2 Antigen Test Kit	Hangzhou Testsea Biotechnology Co Ltd (China)	10-15	300	300	55
9	InnoScreen COVID-19 Antigen Rapid Test Device	Innovation Scientific Pty Ltd (Australia)	15	400	400	100
10	LYHER Novel Coronavirus (Covid-19) Antigen Test Kit (Colloidal Gold)	Hangzhou Laihe Biotech Co Ltd (China)	15	250	250	100

REFERENCES

1. Deerain JM, Tran T, Batty MB, Yoga Y, Druce J, Mackenzie C, et al. Assessment of twenty-two SARS-CoV-2 rapid antigen tests against SARS-CoV-2: A laboratory evaluation study. medRxiv. 2021:2021.12.15.21267691.
